# Response of Saudi Population to Strict Preventive Measures against COVID-19

**DOI:** 10.3390/ijerph182413424

**Published:** 2021-12-20

**Authors:** Amro K. Bin Abdulrahman, Khalid A. Bin Abdulrahman, Randa M. Nouh

**Affiliations:** 1 Preventive Medicine, Department of Public Health, Ministry of Health, Riyadh 13317, Saudi Arabia; 2 College of Medicine, Imam Mohammad Ibn Saud Islamic University, Riyadh 13317, Saudi Arabia; kab@imamu.edu.sa; 3 Joint Program for the Saudi Board of Preventive Medicine, Riyadh Field Epidemiology Training Program Training Center, Assistant Agency for Preventive Health, Department of Public Health, Ministry of Health, Riyadh 13317, Saudi Arabia; randanooh11@gmail.com

**Keywords:** COVID-19, preventive measures, hands hygiene, Saudi Arabia, SARS-CoV-2, public health

## Abstract

The Saudi ministry of health (MOH) started the preventive measures very early on before having a single case of COVID-19. There were very few studies regarding the awareness and adherence to the preventive measures against COVID-19 among the Saudi population in the literature. **Objectives:** The study aims to examine the awareness and commitment to the strict Saudi government preventive measures against COVID-19. **Methods:** A cross-sectional online survey targeted Saudi and non-Saudi populations aged 18+ in March–April 2020. The online questionnaire was designed to explore the participant’s sociodemographic data, washing hygiene habits, the general level of awareness regarding COVID-19, and the extent to which they adhere to the government’s strict instructions. **Results:** Out of the 2958 participants in the survey, 23% washed their hands for between 20 and 30 s, 59.6% washed their hands after shaking hands with other people, 67.9% washed their hands after use of other’s utilities, 65.9% had appropriately followed the MOH recommended guidelines for home quarantine and social distancing. People in different age groups differed significantly on their practiced hygienic practices score *p* < 0.001. Respondents’ educational level had converged considerably and positively on their clean proper prevention practices score, f(2838.3) = 15.70. **Conclusion:** The majority of the participants adhere to the strict government instructions regarding COVID-19 as they have to obey the law. Health sector employees measured significantly greater hygienic preventive measures and precautions in comparison to other sectors. More public health efforts should increase hygienic best practice scores to achieve the best outcome.

## 1. Introduction

Since the emergence of the Coronavirus disease 2019 (COVID-19) in Wuhan, China, in December 2019, there have been 250,154,972 confirmed cases of COVID-19, including 5,054,267 deaths, reported on 10 November 2021 to the world health organization (WHO) [1]. Lessons learned from China are that strict preventive measures will reduce the number of new cases and minimize the country’s healthcare system burden [2,3,4,5,6,7]. Such measures include the following three levels: Governmental, Institutional, and Individual levels. Examples of Governmental levels include: isolating the highly contaminated places or even cities, similar to what happened in Wuhan—shutting down the transportation to and from endemic areas around the world, and in addition, a partial or complete curfew. The prohibited face-to-face educational classes postponed or canceled any social events such as sports, conferences, festivals, etc. They were forcing suspected cases among travelers to declare travel history to endemic places. The Saudi government issued penalties for gatherings that violated the precautionary and preventive measures taken by the concerned authorities to confront the Coronavirus pandemic: It includes SAR 10,000 for a family gathering, SAR 15,000 for a non-family gathering, SAR 40,000 for gatherings for social purposes, such as mourning, parties, etc., and SAR 50,000 for any gathering of workers, with the penalty being applied to every person who attends gatherings.

A violation is SAR 5000. Upon repetition, the penalty imposed on the previous time is doubled and amounts to SAR 100,000, and referring the person to the Public Prosecution for the second time to consider his imprisonment according to the legal procedures followed. The application of a penalty of SAR 10,000 is given to anyone who calls for any of the violating gatherings or causes them, and upon repetition, the penalty imposed on the previous time is doubled and reaches SAR 100,000, in addition to referring them to the Public Prosecution when repeating for the second time to consider his imprisonment in accordance with the regular procedures followed.

The Saudi Ministry of Interior stated that the sanctions aim to impose social distancing and organize human gatherings that are a direct cause of the outbreak of the Coronavirus in a manner that ensures preventing its spread and losing control and containing it, noting that whoever violates the instructions of isolation or quarantine shall be punished with a fine of up to SAR 200,000 or imprisonment for a period not exceeding two years or both. In the event of a repetition of the violation, the penalty imposed on the previous time is doubled.

Institutions tried to reduce or completely stop face-to-face meetings and also reduce working hours. They also encouraged good behavior such as e-learning, video conferencing, online work, etc. At the individual level, all people were asked to stay home almost all of the time. They were encouraged to follow the precautions of COVID-19. For example, they washed hands with soap, rubbed or used alcohol-based hand sanitizers, practiced distancing with family and friends, and avoided handshaking [8,9]. The population was asked to self-report suspected symptoms of COVID-19 and seek medical attention if symptoms worsened. Luckily enough, the Saudi Government has learned from the MERS-COV 2012 experience [10,11].

Moreover, the Saudi Ministry of health was ready to deal with such a crisis. It had a very long experience dealing with mass gathering preventive medicine for millions of pilgrims in Makkah and holly mosques international visitors each year. The Saudi ministry of health started arranging preventive measures very early before having a single case. However, on 2 March 2020, the first case of COVID-19 was confirmed by a Saudi adult who traveled to Iran and re-entered Saudi land without declaring it to the health authorities. One of the lessons learned from the previous MERS-COV 2012 is that strict public response to the preventive measure at the individual level is highly recommended.

During the first two months of the pandemic, there were few studies regarding this topic among the Saudi population available in the published literature [12,13,14]. Therefore, the study aimed to measure the awareness and adherence of the Saudi people to the strict government preventive measures against COVID-19.

## 2. Materials and Methods

### 2.1. Study Designs

This is a cross-sectional study conducted during March and April 2020. The study was conducted according to the guidelines of the Declaration of Helsinki and approved by the Institutional Review Board of the Imam Mohammad Ibn Saud Islamic University IRB committee; project number 11-2020 dated 1 April 2020.

### 2.2. Participants and Sampling

Inclusion criteria: the study targeted Saudi and non-Saudi people aged above 18 years old living in Saudi Arabia.

Exclusion criteria: those under 18 years old and those not living in Saudi Arabia.

The participant’s contact information was obtained through the official Saudi Telecom database, which clearly explained that their communication was purely for scientific purposes. All data of their identities will remain confidential. A cluster sample covering the whole regions in Saudi Arabia followed by a simple random sampling of those randomly selected participants from different parts of Saudi Arabia was applied using a computer generating system to ensure it represented the whole country.

### 2.3. Study Questionnaire

The questionnaire is composed of 19 questions designed to explore the participant’s sociodemographic data, washing hygiene habits, the general level of awareness regarding COVID-19, and the extent to which they adhere to the government’s strict instructions. The questionnaire also looked at their primary source of information regarding COVID-19, the level of stress, and the core symptoms of depression. They were asked these questions based on their current behavior, aiming to minimize possible recall bias. Only one question was a dichotomous response (yes/no), whereas the rest were tick-all-that applies or Likert scale questions.

The questionnaire was subjected to a pilot study of 25 participants. Further improvement and rephrasing were carried out accordingly. The participants were informed about the purpose of the study. Instructions regarding the questionnaires were provided to volunteering participants. The confidentiality of information was also ensured. Once participants voluntarily signed the informed consent, they were requested to fill in the study questionnaire. The online questionnaire was sent via email to 3000 randomly selected populations who met the inclusion criteria. Each participant has sent text messages with three reminders every other day. The Survey Monkey program was used to have a more straightforward presentation of the participant’s questions [15].

### 2.4. Sample Size Calculation

The sample size was calculated using the formula: Z is the standard normal variate, given as 1.96 for 5% type 1 error (*p*-value < 0.05). *p* is the expected proportion in the population (99% of population-based on the previous study = (0.99). d is the margin of error (5% = 0.05). The sample size calculation was based on a previous study [16] The formula is: necessary sample size = (Z-score)2 × *p* × (1 − *p*)/(margin of error)2 = (1.96 × 2 × 0.99 [0.99])/(0.05)2 = (3.8416 × 0.9801)/0.0025 = 3.7651/0.0025 = 1506. The estimated sample size was 1506.

### 2.5. Statistical Data Analysis

The means and standard deviations were used to describe the continuously measured variables and the frequencies and percentages for the categorically measured variables. The Kolmogorov–Smirnov statistical test of normality and the histograms were used to assess the statistical normality of the measured continuous variables and the Levene’s test of equal variance for testing the homogeneity of statistical variance assumption. The multiple-response dichotomy analysis was applied to describe the questions measured with multiple options, such as the hygienic practices and knowledge of COVID-19 disease transmission and prevention methods. People’s hygienic and precautionary practices were measured with ‘tick all that applies. Some of those practices were evidence-informed, and others were not evidence-informed infection prevention measures. We computed the total (sum) of the good practices people have performed to prevent the infection, yielding a total hygienic practices score bounded between 0 and 17 points (or marks). The mean score in the results section corresponds to the mean number of the good hygienic and preventive practices they have performed to prevent the infection. In simpler words: we counted the number of good practices they selected out of a list. The multiple response dichotomies analysis was described in the statistical data analysis section. It was applied to all questions measured with more than one option (tick all that applies’ questions).

The bivariate Pearson’s (r) test of correlation was used to assess the association between metric variables and the independent samples t-test, and the one-way ANOVA test was used to determine the statistical significance of the mean differences on peoples measured knowledge and hygienic preventive activities across the levels of people’s measured sociodemographic characteristics. The multivariate linear regression analysis was applied to people’s hygienic preventive/precautionary practice scores to assess the combined and individual associations between people’s sociodemographic characteristics and their knowledge with their mean measured proper prudent practices undertaken during the pandemic. SPSS IBM V20 (IBM, Armonk, NY, USA) was used for the data analysis, and the statistical significance alpha level was considered as 0.050. Excel was used for creating figures and depictions.

## 3. Results

Out of the 3000 invited population, 2598 (86.6%) people residing in Saudi Arabia had enrolled themselves electively into the study. The yielded data analysis results for their sociodemographic characteristics are displayed in Table 1.

### People Practiced Hygienic and Preventive Precautionary Measures during the Pandemic

The respondents were asked several questions measuring their sanitary practices, actions, behaviors, and social distancing activities. The resulting descriptive analysis findings are displayed, and the best correct practices based on medical evidence are marked with an (*) in Table 2.

The findings from the analysis showed that 23% of the respondents had correctly been washing their hands for 20–30 s, and another 22.8% had also been scrubbing their hands appropriately for 30–40 s. Nonetheless, a few respondents advised they clean their hands for 40 s when washing their hands.

Moreover, the resulting findings showed that 59.6% of them washed their hands after shaking hands with other people, and 67.9% washed their hands after using others’ utilities. However, 52.9% advised they washed hands before entering their homes and 88% after leaving the bathroom/toilets, 84.5% washed their hands before eating and 84.5% after eating, and another 63% washed their hands after touching other people’s belongings.

They were also asked to rate themself for how often they had used the medical alcohol-based scrub gel for sanitizing their hands, and the respondents overall mean collective rating of alcohol gel use was rewarded with 3.73 points out of 5, but 4% of them advised they had never used the alcohol gel. Another 9.3% of them had used the alcohol gel rarely, 30.8% had used it sometimes, but 21.2% had used the alcohol-based gel usually to sanitize their hands, and most of them, 34.6%, however, had always used the alcohol-based gel as a hand sanitizer.

The respondents were asked to select all occasions they had found themselves leaving their homes during the lockdown (stay at home) issued by the government during the active phase of the COVID-19 pandemic. Most of them, 75.1%, had left their homes for shopping and 44.4% for buying necessary medications and hygienic sanitizers essential for them and their houses. Next, the respondents were asked to select a list of all that applied to them of their actions to protect them from contracting the COVID-19 disease during the pandemic. More than 46.0% of the respondents had correctly kept a physical distance between them and other family members during their daily interaction; another 43.5% had rightly avoided handshaking and physical contact with suspected family members with signs of the flu. However, 65.9% advised they had appropriately followed the Ministry of Health recommended guidelines for home quarantine and social distancing. Moreover, 37.4% reported that they had worked online or via distance with their clients, 54.7% participated in online shopping, 43.1% browsed the internet, and 15.3% of them were enrolled in voluntary online work. Overall, proper hygienic practices were measured with 9.1 points out of 18 maximum points, SD = 3.1 points, which in general means the best hygienic practices score is equivalent to 50.5% out of the maximum 100% best practices points when expressed as a percentage out of hundred percent, which is indicative of reasonable but substantial hygienic preventive measures and compliance with guidelines practiced by the respondents on average.

To arrive at a better understanding of why people may have differed on the hygienic preventive measures undertaken during the pandemic, the analysis went further by analyzing their mean COVID-19 precautionary practices score and the statistically significant mean differences across the levels of their measured characteristics.

**Figure 1:**
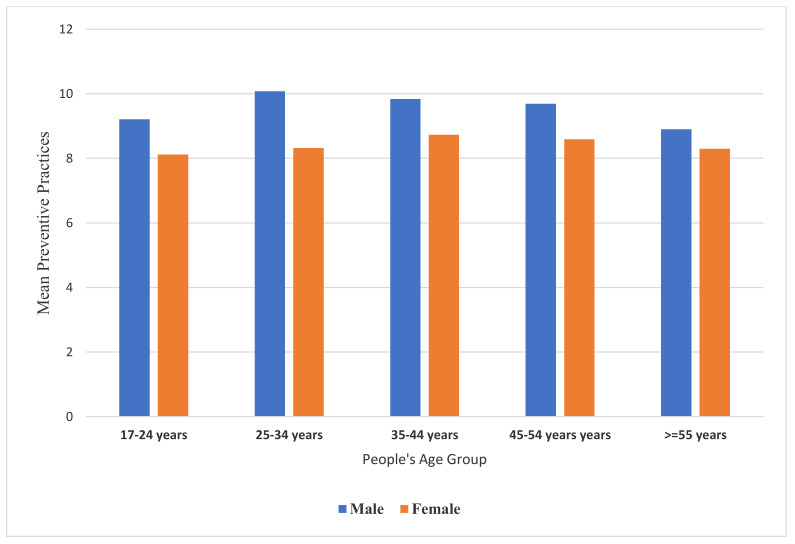
The association between people’s age with their mean practiced COVID-19 preventive measures for both genders.

The resulted findings suggested that the females had significantly lower mean hygienic preventive measures against the COVID-19 (M = 8.38, SD = 3.10) than males (M = 9.72, SD = 3.00), *p* < 0.001 according to an independent samples t-test, but a Welch’s adjusted one-way ANOVA test suggested that people in different age groups differed significantly on their practiced hygienic practices score, f(4871.5) = 6.24, *p* < 0.001 and a Games–Howell post hoc pairwise comparison test showed that people aged 18–24 years had practiced significantly less correct preventive measures (M = 8.52, SD = 3.23) than those aged 25–34 years, *p* = 0.008. Furthermore, those aged 18-24 showed significantly lower preventive measures than people aged 35–44, *p* < 0.001, but also they (aged 18–24 years) showed significantly lower hygienic preventives than those people aged 45–54 years (M = 9.36, SD = 3.77); however, those aged 18–24 years did not differ considerably from those aged ≥ 55 years on their respective hygienic preventive measures, *p* = 0.928. The other pairwise comparisons between people in different remaining age groups suggested they may not necessarily differ significantly in their corresponding hygienic mean practice scores. Figure 1 demonstrates that the young and the very old tended to practice less sanitary preventive measures than different age groups.

Peoples’ marital state did not correlate significantly with their hygienic practices despite the slight difference between married and single people on their mean hygienic preventive precautionary measures. Unsparingly, peoples educational level had converged significantly and positively on their clean proper prevention practices score, f(2838.3) = 15.70, according to Welch’s corrected one-way ANOVA test, and a follow up pairwise comparison test showed that people educated with high school or lower education measured significantly lower preventive hygienic practices (M = 8.66, SD = 3.26) than people educated with a college/university degree (M = 9.10, SD = 3.10), *p* < 0.001. Furthermore, those with high school or lower education showed significantly less preventive hygienic practices than those educated with higher studies (M = 9.81, SD = 2.92), *p* < 0.001, and in addition, those with higher education showed significantly greater hygienic preventive and precautionary measures than those with a college/university degree, *p* < 0.001.

According to a one-way ANOVA test, people from various Saudi Kingdom regions differed significantly on their mean hygienic preventive practices against COVID-19. A post hoc pairwise analysis showed that only Eastern Provinces practiced significantly greater mean precautionary hygiene than those from Western Saudi Provinces, *p* = 0.006. However, the other people from different regions did not differ on the sanitary precautions they may have undertaken. Furthermore, people working in various job sectors showed significantly different mean hygienic precautionary measures, f(8262.9) = 28.8 according to Welch’s One-way ANOVA test, *p* < 0.001, and a post hoc pairwise comparison between those people on their mean hygienic practices showed that the housewives and retired people had significantly lower mean sanitary preventive measures than did each of the people working in the health sector and educational sector as well as the governmental services and military services, *p* < 0.001 each, respectively. Furthermore, the housewives and retired people in unpaid jobs showed significantly lower mean hygienic practices and preventive measures than those who were self-employed, *p* = 0.034. The pairwise test suggested that students have considerably lower mean hygienic practices than those in private and health sectors, *p* < 0.001, respectively. The students also measured significantly fewer precautions than those working in the military and governmental sectors, *p* = 0.001 each, respectively. The post hoc analysis showed that the health sector employed significantly greater hygienic preventive measures than people working in private, education, and government sectors, *p* < 0.001 each, respectively. The health sector employed people who measured significantly greater sanitary precautions than self-employed people, *p* = 0.008. The private sector used people from hand-measured significantly greater mean hygienic practices than people in educational jobs. Still, the other pairwise comparisons between people in different job sectors yielded no statistically significant mean differences.

Table 3 quantifies people’s compliance level with the governmental guidelines and recommendations. The respondents were asked to select out of a list of actions and suggestions that they had applied on a regular basis; 87.1% of people washed their hands effectively, another 78.8% of them advised they had complied with avoidance of handshaking, 91% of them had covered their face with a tissue when sneezing, 77.6% of them kept a sufficient physical distance of one meter when interacting with others, 77.2% of the respondents advised they isolated themselves once they had suspicious signs and symptoms of the COVID-19 disease, 77.2% of the respondents advised they had not used other people’s utilities, 75.6% performed all their five prayers at home, 79.7% avoided sharing inaccurate information with others, 64.5% advised they believed donning gloves was not an effective COVID-19 disease transmission preventive and that they may lead to a false sense of cleanliness if not changed regularly, and 56.8% of the respondents advised they had cooked vegetables and foods properly to sterilize them.

The multivariate linear regression analysis indicated that female residents practiced significantly lower preventive hygienic measures than males, *p* < 0.001 Table 4. Accounting for other predictors in the analysis model, demographic factors such as age, nationality, marital state, educational levels, and province location did not converge significantly on their hygienic preventive measures score; however, healthcare practitioners compared to non-healthcare practitioners showed significantly greater sanitary preventive measures on average, *p* = 0.001, when considering the other predictors as accounted for. Moreover, people’s knowledge score on COVID-19 had converged significantly and positively on their hygienic preventive measures practiced during the pandemic lockdown, *p* < 0.001, when considering the other predictors as accounted for.

In addition, people working in the private sector showed significantly higher mean hygienic practices than the others (housewives, retired, students, and people working in educational fields), *p* < 0.001. Those people working in the health sector also practiced significantly greater prevention than others, *p* < 0.001; likewise, the people working in the governmental and military sectors, as well as self-employed people, practiced significantly greater hygiene than people in different sectors outside of the analysis model mentioned earlier, *p* < 0.001 each respectively. People’s perceived worry level from the COVID-19 did not correlate significantly with their hygienic practices; however, *p* = 0.186 considered the other predictors in the analysis model.

## 4. Discussion

To our knowledge, this is one of the few studies in Saudi Arabia that measured how Saudi people responded to the strict government instructions in the early months of the crisis with such a significant sample size.

The majority of Saudi people followed the government’s strict instructions toward COVID-19 prevention and instructions. They washed their hands effectively, avoided handshaking, covered their face with a tissue when sneezing, kept a sufficient physical distance of one meter when interacting with others, isolated themselves once they had suspicious signs and symptoms of the COVID-19 disease, had not used other people’s items, performed all their five prayers at home, avoided sharing inaccurate information with others, and believed wearing gloves was not an effective COVID-19 disease transmission preventive and that they may lead to a false sense of cleanliness if not changed regularly. This might be explained by the country system’s nature, as the king has strict laws with all government bodies’ support. Those who do not follow will either pay fines or will face penalties. Therefore, it was easier to control people’s behavior regarding the high percentage of shopping. They might be willing to buy more supplies and prepare for the Muslim holy events that require shopping, such as the month of Ramadan and Eid ceremony preparations. The previous finding was supported by other studies [12,13,14,15,16,17].

The majority of the participants wash their hands with soap and water with hand rubbing. This finding was supported by other studies conducted in Saudi Arabia and other countries [17,18,19,20].

It is not surprising that there was an increase in washing hands with soap and water rubbing after leaving the bathroom. Furthermore, many people use hand sanitizers more frequently. This was also supported by previous studies [18,21]

Overall proper hygienic practices were measured with 9.1 points out of 18 maximum points; SD = 3.1 points. This overall mean hygienic best practice score is equivalent to 50.5% out of a maximum of 100%.

This moderate level of knowledge might be reasonable as the crisis had just begun at that time. Nevertheless, more public health efforts should increase the hygiene best practice score to achieve the best outcome.

The current study showed that the females measured significantly lower mean hygienic precautionary against the COVID-19 than males. This result could be explained by the fact that females tended to stay at home more often than males during the beginning of the crisis. Therefore, they might have a false sensation of being clean and safe from the infection while staying at home. Nevertheless, other Saudi Arabia and Portugal studies have found that females showed a better performance than males [22,23]. The previous Saudi survey concluded that women were more compliant with the WHO public health COVID-19 prevention advice than men, decreasing the chances of COVID-19 infection.

People aged 18–24 years had practiced significantly less correct preventive measures than older age groups. This result could be justified by acknowledging that some age groups are prone to caring less about hygiene as they may not comprehend the consequences of these behaviors. Some of them might think they are too young to become sick, and even if they become ill, they have young bodies, and their immunity will do the rest of the work. This result was similar to a study conducted in Ghana [24].

Those over 55 years old also reported practicing fewer preventive measures. The more aging population might not be exposed to social media campaigns than younger people. Therefore, proper care and support should be provided to them.

The participants with higher education levels had higher preventive hygienic practices and vice versa. This result is logical as when education improves, health awareness improves, and people are more likely to be health literate. The same applies to the lower education level. Health education and public health may not be priorities as they may not appreciate its value [23,25].

Eastern Provinces had measured significantly greater mean hygienic precautions than Western Saudi Provinces, *p* = 0.006. This might be explained by the fact the western regions tend to have more cultural diversity from the increased number of immigrants each year. As usual, they move to this region near the holy mosque in Makkah. However, it is different from eastern provinces as the people might be influenced by countries such as Bahrain and modern cities such as Jubail and Dharan. This may all reflect the quality of life, as those who live there might be more educated.

The housewives and retired people in unpaid jobs measured significantly lower mean hygienic practices and preventive measures than those who are self-employed or work in governmental sectors. These results could be justified because governmental sectors have vast efforts and strict protocols regarding COVID-19 instructions such as hand hygiene, wearing masks, social distancing, and prohibiting handshaking. Therefore, compared to retired people and housewives, they are not exposed to such initiatives.

The study finding suggested that pre-university students had measured significantly lower mean hygienic practices. The youth age group might be less knowledgeable and less experienced in life. Some might be even less responsible. Therefore, more efforts should be made to educate them via their schools [23,24]. Health sector employees measured significantly greater hygienic preventive measures and precautions in comparison to other sectors. This result is logical, as health education is part of their training, and they are considered role models to society at large [16].

Moreover, people’s knowledge score on COVID-19 had significantly and positively converged on the hygienic preventive measures they had practiced during the pandemic lockdown. The lockdown indeed forces many people to be more aware of what is going on worldwide. Thus, it increases the chances of following hygienic preventive measures and precautions [24].

People working in the private sector had measured significantly higher mean hygienic practices than the others (housewives, retired, students, and people working in educational fields). The private sectors are strict with their employees if they make any mistakes. The governments would give them penalties for not following the law. Therefore, it explains the high level of adherence and awareness [16].

## 5. Limitations

The study participants might have been exposed to recall bias. Nevertheless, this was minimized by asking about their current behavior. Some factors might affect the results of the study. However, this was managed by a different statistical test to adjust these factors and provide a more accurate result. The cross-sectional design of our analysis does not allow for a causal interpretation of our findings. Therefore, other studies that have more vital causality are recommended.

## 6. Conclusions

The sample participants in Saudi Arabia have moderate but substantial hygienic preventive and compliance with guidelines practiced. The majority of the participants adhere to the strict government instructions regarding COVID-19 as they have to obey the law. Health sector employees measured significantly greater hygienic preventive measures and precautions in comparison to other sectors.

More public health efforts should increase hygienic best practice scores to achieve the best outcome. Public health efforts should be focusing more on females, students, housewives, retired people, and those aged 18–24 and above 55 years old.

## Figures and Tables

**Table 1:** Descriptive analysis of the respondent’s sociodemographic characteristics. *n* = 2598.

	Frequency	Percentage
Sex		
Male	1390	53.5
Female	1208	46.5
Age		
18–24 years	420	16.2
25–34 years	1015	39.1
35–44 years	602	23.2
45–54 years	351	13.5
≥55 years	210	8.1
Nationality		
Saudi	2131	82
Non-Saudi	467	18
Marital state		
Never Married	728	28
Ever Married	1870	72
Educational Level		
High school or less	550	21.2
College/University Degree	1678	64.6
Higher studies	370	14.2
Residence Location		
Eastern Provinces	412	15.9
Western Provinces	1107	42.6
Northern Provinces	149	5.7
Southern Provinces	152	5.9
Central Provinces including Riyadh	778	29.9
Occupation type sector		
Private sector	687	26.4
Health sector	251	9.7
Educational sector	192	7.4
Governmental service	288	11.1
Non-Profit charitable	18	0.7
Military sector	90	3.5
Self-employed/Free Jobs	52	2
Student	307	11.8
Housewife and/Retired	713	27.4
Type of house		
Other residential places	33	1.3
Apartment/house	1404	54
Villa	998	38.4
Private Palace	18	0.7
A residential compound	59	2.3
Motel/mansion	5	0.2
Shared house	3	0.1
Conventional house	73	2.8
Residential Annex	5	0.2
Are you a healthcare worker?		
No	2203	84.8
Yes	395	15.2

**Table 2:** The respondent’s hygienic and social distancing practices during the pandemic period. *n* = 2598.

	Frequency	Percentage
How much time do you spend scrubbing your hands with soap and water while washing		
I never counted the time	175	6.7
<10 s	207	8
10–20 s	833	32.1
20–30 s *	601	23.1
30–40 s *	593	22.8
>40 s	189	7.3
When do you wash your hands?		
After shaking hands *	1548	59.6
After the use of other utilities *	1764	67.9
Before entering home *	1375	52.9
After leaving the toilet *	2285	88
Before eating *	2186	84.1
After eating *	2196	84.5
After touching doorknobs and other surfaces *	1636	63
I never wash	13	0.5
How often do you use alcohol gel-based rubs, mean (SD)		3.73 (1.15)
I never use alcohol rubs	103	4
Rarely	242	9.3
Sometimes	801	30.8
Usually *	552	21.2
Always *	900	34.6
On what occasions did you find yourself leaving home when required to stay home during the COVID-19 pandemic?		
To buy coffee	56	2.2
For sports	81	3.2
For work *	766	29.8
For a short picnic/stroll	22	0.9
For social gatherings of 10 or more persons	9	0.4
For buying groceries and/shopping *	1930	75.1
For buying necessary medications *	1141	44.4
For smoking/buying cigarettes	58	2.3
If I feel sick and need medical care *	708	27.6
Did not leave home at all	563	21.9
What did you do during your stay at home to protect yourself from COVID-19?		
I kept a distance between family members and me *	1131	46.4
I avoided handshaking and physical contact with family members *	1060	43.5
I isolated myself in a separate room	304	12.5
I welcomed friends and relatives to my house	42	1.7
I called the private teacher to my house	11	0.5
I received the technicians and maintenance workers in my house	77	3.2
I let the massage therapist come to my house	4	0.2
I let the hairdresser and/barber come to my home	7	0.3
I followed the updated recommendations from the MOH *	1607	65.9
I work online/via distance	911	37.4
I did shopping online	1334	54.7
I browsed and learned online	1051	43.1
I did voluntary work online	372	15.3

* The resulting descriptive analysis findings are displayed, and the best correct practices based on medical evidence

**Table 3:** Which of the below recommendations by MOH did you comply with? *n* = 2598.

I washed hands thoroughly with soap and water for 40 s after leaving the toilet or touching suspicious/unclean subjects	2260	87.1
Avoided handshaking even my family members	2045	78.8
I covered my face with a tissue while sneezing/sneezing away toward my shoulders	2336	90
I kept a distance of one meter between me and others, including family members	2014	77.6
When I had suspicious flu symptoms, I covered my face and isolated myself in a separate place	1943	74.9
I avoided the use of the utilities of others, even my family members	2003	77.2
I did all my five prayers at home	1962	75.6
I avoided sharing unclear/inaccurate COVID-19 information with others over social media	2068	79.7
I stay at home until it is permissive by the government directives	2341	90.2
I believe gloves do not protect against COVID-19 transmission and/give a false sense of cleanliness	1673	64.5
I cooked vegetables and foods properly	1473	56.8

**Table 4:** Multivariate linear regression analysis of the patient’s hygienic practices score during the COVID-19 pandemic. *n* = 2597.

		95% C. I for Beta Coefficient			
	Beta Coefficient	Lower Bound	Upper Bound	t-Value	*p*-Value
(Constant)	3.261	2.332	4.189	6.885	<0.001
Sex = Female	−0.894	−1.143	−0.645	−7.040	<0.001
Age group	−0.005	−0.123	0.112	−0.089	0.929
Nationality = Saudi	−0.106	−0.410	−0.198	−0.683	0.495
Educational Level	0.040	−0.161	0.241	0.391	0.696
Married	−0.180	−0.473	−.113	−1.203	0.229
Location	−0.038	−0.110	0.035	−1.017	0.309
Healthcare worker = Yes	0.692	0.299	1.085	3.455	0.001
Worry Level from COVID-19 mean score	0.027	−0.013	0.067	1.323	0.186
Knowledge score on COVID-19 disease	0.497	0.434	0.560	15.543	<0.001
Work = Private sector	0.936	0.644	1.229	6.284	<0.001
Works in the health sector	1.434	0.950	1.917	5.814	<0.001
Works in governmental services sector	0.948	0.561	1.336	4.797	<0.001
Works in military services	1.224	0.594	1.854	3.811	<0.001
Self-employed	0.779	−0.010	1.569	1.936	0.053

Dependent variable = proper hygienic and social distancing behaviors score. Model overall R = 42.4%, model adjusted R-squared = 17.5, model overall statistical significance: f (14,2583) = 40.34, *p* < 0.001.

## Data Availability

Data is available on request due to restrictions on the participants’ privacy.

## References

[1] WHO Coronavirus (COVID-19) Dashboard. https://COVID-19.who.int.

[2] Pan Y., Fang Y., Xin M., Dong W., Zhou L., Hou Q., Li F., Sun G., Zheng Z., Yuan J. (2020). Self-Reported Compliance With Personal Preventive Measures Among Chinese Factory Workers at the Beginning of Work Resumption Following the COVID-19 Outbreak: Cross-Sectional Survey Study. J. Med. Internet Res..

[3] Zhang L., Shen M., Ma X., Su S., Gong W., Wang J., Tao Y., Zou Z., Zhao R., Lau J. (2020). What is required to prevent a second major outbreak of the novel coronavirus SARS-CoV-2 upon lifting the metropolitan-wide quarantine of Wuhan city, China. medRxiv.

[4] Esposito S., Principi N., Leung C.C., Migliori G.B. (2020). The universal use of face masks for success against COVID-19: Evidence and implications for prevention policies. Eur. Respir. J..

[5] Maharaj S., Kleczkowski A. (2012). Controlling epidemic spread by social distancing: Do it well or not at all. BMC Public Health.

[6] Kwok K.O., Li K.K., Chan H.H.H., Yi Y.Y., Tang A., Wei W.I., Wong S.Y.S. (2020). Community Responses during Early Phase of COVID-19 Epidemic, Hong Kong. Emerg. Infect. Dis..

[7] Wang C., Pan R., Wan X., Tan Y., Xu L., Ho C.S., Ho R.C. (2020). Immediate Psychological Responses and Associated Factors during the Initial Stage of the 2019 Coronavirus Disease (COVID-19) Epidemic among the General Population in China. Int. J. Environ. Res. Public Health.

[8] Halperin D.T., Hearst N., Hodgins S., Bailey R.C., Klausner J.D., Jackson H. (2021). Revisiting COVID-19 policies: 10 evidence-based recommendations for where to go from here. BMC Public Health.

[9] World Health Organization (2020). Recommendations to Member States to Improve Hand Hygiene Practices to Help Prevent the Transmission of the COVID-19 Virus. https://www.who.int/publications-detail/recommendations-to-member-states-to-improve-hand-hygiene-practices-to-help-prevent-the-transmission-of-the-COVID-19-virus.

[10] Al-Tawfiq J.A., Memish Z.A. (2020). Middle East Respiratory Syndrome Coronavirus and Severe Acute Respiratory Syndrome Coronavirus. Semin. Respir. Crit. Care Med..

[11] Al-Tawfiq J.A., Zumla A., Memish Z.A. (2014). Coronaviruses: Severe acute respiratory syndrome coronavirus and Middle East respiratory syndrome coronavirus in travelers. Curr. Opin. Infect. Dis..

[12] Abolfotouh M.A., Almutairi A.F., Banimustafa A., Hagras S.A., Al Jeraisy M. (2021). Behavior Responses and Attitude of the Public to COVID-19 Pandemic During Movement Restrictions in Saudi Arabia. Int. J. Gen Med..

[13] Obeid D.A., Alhamlan F.S., Al-Qahtani A.A., Al-Ahdal M.N. (2020). Containment of COVID-19: The unprecedented response of Saudi Arabia. J. Infect. Dev. Ctries..

[14] Temsah M.H., Alhuzaimi A.N., Alamro N., Alrabiaah A., Al-Sohime F., Alhasan K., Kari J.A., Almaghlouth I., Aljamaan F., Al-Eyadhy A. (2020). Knowledge, attitudes and practices of healthcare workers during the early COVID-19 pandemic in a main, academic tertiary care centre in Saudi Arabia. Epidemiol. Infect..

[15] SurveyMonkey, the World’s Leading Survey Platform. https://www.surveymonkey.com.

[16] Al-Rasheedi M., Alhazmi Y., Mateq Ali A., ALrajhi M., Alharbi N.S., Alsuhaibani S., Mohammed A., Alharbi G. (2021). Public and healthcare providers awareness of Coronavirus (COVID-19) in Qassim Region, Saudi Arabia. Saudi J. Biol. Sci..

[17] Zakout Y.M., Khatoon F., Bealy M.A., Khalil N.A., Alhazimi A.M. (2020). Role of the Coronavirus Disease 2019 (COVID-19) pandemic in the upgrading of personal hygiene. A cross-sectional study in Saudi Arabia. Saudi Med. J..

[18] Abdulrahman A.K.B., Abdulrahman K.A.B., Almadi M.K., Alharbi A.M., Mahmoud M.A., Almasri M.S. (2019). Do various personal hygiene habits protect us against influenza-like illness?. BMC Public Health.

[19] Al-Wutayd O., Mansour A.E., Aldosary A.H., Hamdan H.Z., Al-Batanony M.A. (2021). Handwashing knowledge, attitudes, and practices during the COVID-19 pandemic in Saudi Arabia: A non-representative cross-sectional study. Sci. Rep..

[20] Mahdi H., Alqahtani A., Barasheed O., Alemam A., AlHakami M., Gadah I., Alkediwi H., Alzahrani K., Fatani L., Dahlawi L. (2020). Hand Hygiene Knowledge and Practices among Domestic Hajj Pilgrims: Implications for Future Mass Gatherings Amidst COVID-19. Trop. Med. Infect. Dis..

[21] Gharpure R., Miller G.F., Hunter C.M., Schnall A.H., Kunz J., Garcia-Williams A.G. (2020). Safe Use and Storage of Cleaners, Disinfectants, and Hand Sanitizers: Knowledge, Attitudes, and Practices among U.S. Adults during the COVID-19 Pandemic, May 2020. Am. J. Trop. Med. Hyg..

[22] Alshammary F., Siddiqui A.A., Amin J., Ilyas M., Rathore H.A., Hassan I., Alam M.K., Kamal M.A. (2021). Prevention Knowledge and Its Practice Towards COVID-19 Among General Population of Saudi Arabia: A Gender-based Perspective. Curr. Pharm. Des..

[23] Alves R.F., Samorinha C., Precioso J. (2020). Knowledge, attitudes and preventive behaviors toward COVID-19: A study among higher education students in Portugal. J. Health Res..

[24] Apanga P.A., Kamal Lettor I.B., Akunvane R. (2020). Practice of COVID-19 Preventive Measures and Its Associated Factors among Students in Ghana. Am. J. Trop. Med. Hyg..

[25] Zhong B.L., Luo W., Li H.M., Zhang Q.Q., Liu X.G., Li W.T., Li Y. (2020). Knowledge, attitudes, and practices towards COVID-19 among Chinese residents during the rapid rise period of the COVID-19 outbreak: A quick online cross-sectional survey. Int. J. Biol. Sci..

